# Supporting Nature-Based Solutions via Nature-Based Thinking across European and Latin American cities

**DOI:** 10.1007/s13280-023-01920-6

**Published:** 2023-09-26

**Authors:** Geovana Mercado, Tom Wild, Jaime Hernandez-Garcia, Mariana D. Baptista, Martina van Lierop, Olivia Bina, Andy Inch, Åsa Ode Sang, Arjen Buijs, Cynnamon Dobbs, Alexis Vásquez, Alexander van der Jagt, Fabio Salbitano, Roberto Falanga, Juan David Amaya-Espinel, Mafalda de Matos Pereira, Thomas B. Randrup

**Affiliations:** 1 Department of Landscape Architecture, Planning and Management, Swedish University of Agricultural Sciences, Box 190, 234 22 Lomma, Sweden; 2 Department of Landscape Architecture, Faculty of Social Sciences, University of Sheffield, Sheffield, UK; 3 School of Architecture and Design, Pontificia Universidad Javeriana, Bogotá, Colombia; 4 TUM School of Life Sciences Weihenstephan, Technical University of Munich, Munich, Germany; 5 Instituto de Ciências Sociais, Universidade de Lisboa, Lisbon, Portugal; 6 Present Address: Department of Urban Studies and Planning, University of Sheffield, Western Bank, Sheffield, S102TN UK; 7 Forest and Nature Conservation Policy Group, Wageningen University, Wageningen, The Netherlands; 8 Department of Natural Resources and the Environment, University of Connecticut, Storrs, USA; 9 University of Chile, Santiago, Chile; 10 The Urban Institute School of Energy, Geoscience, Infrastructure and Society Heriot-Watt University Edinburgh, Edinburgh, UK; 11 Department of Agriculture, Food, Environment and Forestry (DAGRI), University of Florence, Florence, Italy; 12 Facultad de Estudios Ambientales y Rurales, Pontificia Universidad Javeriana Bogotá D.C., Bogotá, Colombia

**Keywords:** Climate change, Nature-Based Solutions, Nature-Based Thinking, Urban governance

## Abstract

Nature-Based Solutions concepts and practices are being used worldwide as part of attempts to address societal challenges but have also been criticised for not dealing with deeper transformations needed to face urgent issues including biodiversity loss, climate change and inclusion. In this paper, we explore how an inclusive, integrated and long-sighted approach, emphasising a more radical integration of nature within cities, might support the transformations needed to endure major contemporary challenges. Addressing important emerging critiques of Nature-Based Solutions, we consider the potential of a more incisive form of *Nature-Based Thinking* (NBT) in cities, based on more holistic perspectives. The paper draws on a reflective and iterative research process that engaged both the research and practice communities through a symposium and a series of futures workshops that together explored the potential of NBT to develop future nature-cities relations in Europe and Latin America. The results of the reflective process suggest that notions of *nature with people*—not for people— new organisational structures, and the intention and capacity to apply long-term perspectives, are needed when planning for NBS interventions aimed at sustainable urban development. This includes developing a cultural-structural change based on new and inclusive understandings of human–nature relations, and novel governance paradigms that allow cross-sectoral coordination and engagement of local stakeholders beyond formal organisational structures.

## Introduction

The implementation of Nature-Based Solutions (NBS), defined early on as “*solutions that are inspired and supported by nature, which are cost-effective, simultaneously provide environmental, social and economic benefits and help build resilience”* (EC 2022) has gained traction to cope with several contemporary societal challenges, such as climate change and biodiversity loss (Feyisa et al. 2014; IPCC 2014; Skoulika et al. 2014; Young et al. 2019). NBS have been tested and used in diverse contexts from biodiversity conservation (TEEB 2010) to engineered stormwater solutions in urban scenarios (Wendling & Holt 2020) and could promote urban re-naturalisation processes as well as reducing climate risks and adding multiple benefits for highly populated areas (Wickenberg et al. 2021). Hence NBS have become central to attempts to overcome urban challenges and scholars call for the need to include them in policy making, planning and management whilst also helping governments, NGOs and the private sector to promote human wellbeing (Cohen-Shacham et al. 2019; Ferreira and Ribeiro 2020; Wickenberg et al. 2021). A recent review of NBS publications developed a list of key characteristics which state that NBS should (i) be inspired and powered by nature, (ii) address (societal) challenges, (iii) provide multiple services/benefits and (iv) be effective and economically efficient (Sowinska-Swierkosz and Garcia 2022; see also Dorst et al. 2019). However, even as scholarly and policy interest in NBS has risen exponentially (Wild et al. 2020), ecological, social and economic crises have intensified in scale and urgency, generating important questions about the adequacy of NBS, as originally conceived, as a response to pressing societal challenges.

The problematic idea of ‘solving’ the challenges posed by urban development via NBS risks reproducing or exacerbating instrumentalised views of nature as external to the social realm or as a resource to be exploited (Schröter et al. 2014; Eggermont et al. 2015). Critics have argued that the ambiguity of the NBS concept could make it vulnerable to co-option, and to be used as a greenwashing strategy, which has led to interpreting the NBS concept as a ‘dangerous distraction’, which impels tangible structural changes (Melanidis and Hagerman 2022). In this conception, NBS can be seen as part of an eco-modernist or ‘green growth’ approach that disavows the wider structural drivers of change and transformations required to respond to climate and nature emergencies. Linked to this, critics question the anthropocentric and instrumental conceptions of nature assumed by NBS (Pereira and Bina 2020; Tozer et al. 2020; Maller 2021). This is evident in the idea that nature-based interventions offer ‘solutions’ to a wide range of challenges and priorities. For some critical voices, this idea is rooted in the very forms of Capitalist modernity, Western instrumental rationality and faith in technological progress, which created the crises of the Anthropocene in the first place (e.g., Morton 2016). Despite an explicit emphasis within NBS on moving beyond grey engineering within cities, the idea that the ‘wicked problems’ found in complex, open systems can be ‘solved’ has long been critiqued (Rittel and Webber 1973, p.160). From this viewpoint, NBS may be seen as imbued with significant ontological and epistemological assumptions which may constrain rather than enable wider processes of change (Pereira and Bina 2020). For example, the prevailing emphasis on technocratic solutionism in urban development has been shown to dominate narratives and imaginaries (Bina et al. 2020) and may therefore limit the possibility of thinking about cities from radically different perspectives, where human and other-than human relations are central (Bina and Pereira 2021; Maller 2021).

In this paper, we explore what is seen to impede any inclusive and transformative capacity of NBS. Our aim is to examine the potential value of a deeper form of integration of nature in city planning and management, by exploring how a Nature-Based Thinking (NBT) perspective (Randrup et al. 2020; Wild 2020) can broaden, and expand NBS frameworks through incorporating local contexts and realities (Wickenberg et al. 2021). Nature-Based Thinking suggests a need to recognise nature from a human—and more than human—perspective, acknowledging the intrinsic value of nature, as well as proposing a long-sighted and relational approach to nature (Randrup et al. 2020). We present the results of a reflective, iterative process derived from a transdisciplinary symposium and futures workshops, which engaged multiple stakeholders—from practice and research in seven Latin American and European cities—involved in the implementation of urban NBS. The intention is that these results may help to strengthen the use of NBS not just as solutions, but as a holistic approach to address urban societal challenges. In the following sections, we develop our understanding of NBT, present our methodological approach and discuss our results and conclusions.

## The debate on NBS and the urban context

In addition to the more fundamental, conceptual critiques of NBS, it is also widely recognised that the potential attributed to NBS is often not realised in practice, whether by dominant forms of economic development, lack of political will, and/or inadequate planning and management in cities (Kabisch et al. 2016; Qiao et al. 2018; Dorst et al. 2019). There are also vast differences between countries in the Global North (GN) and the Global South (GS) in knowledge, governance, policy and institutional capacity to implement NBS (Dobbs et al. 2019; Breen et al. 2020). Fragmentation of government, including processes of privatisation and organisational restructuring, has exacerbated hierarchical and silo-dominated organisational environments (Randrup and Jansson 2020). This constitutes a problem for urban NBS implementation, which often requires integrated and transdisciplinary coordination across and beyond scalar boundaries (Wickenberg et al. 2021). This can result in persistent mismatches between the different geographical scales at which governments operate, e.g.,: locations where economic investment decisions are made are not always, where the environmental impacts of urbanisation processes are felt (e.g., Bai et al. 2010).

Contemporary research has frequently suggested that a more systemic approach to urban planning and management might enable urban-based governance systems to promote sustainability transitions, economically, socially and ecologically (Bai et al. 2016; Frank et al. 2017; Duminy and Parnell 2020; Wickenberg et al. 2021). Systems-based approaches including cultural, economic and technological perspectives have been proposed to help maximise co-benefits and synergies, and to manage inevitable trade-offs such as the mismatch between urban policies and regional and global environmental issues (see i.e., the vast literature on social-ecological systems (SES) and social-ecological-technological systems (SETS), including: Andersson et al. 2014, 2021; McPhearson et al. 2016, 2022; Wellmann et al. 2023, as well as Bai et al. 2010; Wickenberg et al. 2021). Theoretical interpretations of urban development have often assumed that cities exhibit (eco) systemic behaviours (Bai et al. 2010; Andersson et al. 2014; McPhearson et al. 2016). However, urban science has long grappled with the challenges of analysing complex urban systems (Rittel and Webber 1973; Duminy and Parnell; 2020; Fokdal et al. 2021). Exacerbated by the basic government constraints discussed above, it has proven stubbornly difficult to plan and manage the complexity of cities from a socio-ecological, governance-based perspective (see Elmqvist et al. 2006; Frank et al. 2017; Andersson et al. 2021).

The challenge of governing complex socio-ecological urban systems (Andersson et al. 2014, 2021) is also increased by calls to acknowledge and incorporate diverse ways of knowing and relating to nature. Debates on more inclusive, pluralistic approaches to nature in cities range from the inclusion of relational values, more-than-human approaches, justice in ecosystem services, post development approaches and the life frames approach (Whatmore 2006; Gudynas 2014; O’Conner and Kenter 2019; Langemeyer and Connolly 2020). Scholars have remarked the importance of considering alternative approaches from a plurality of knowledge systems, including principles from non-western worldviews and cultures, in their relations to nature (Dobbs et al. 2019; Pereira and Bina 2020; Tozer et al. 2020; McPhearson et al. 2022). The relevance of indigenous, traditional or ancestral knowledges—understood as place-based and knowledge-practice-value systems—is widely recognised as a precondition for providing resilient and sustainable responses to the climate crisis and nature-based societal challenges (McMillen et al. 2014). In fact, for many First Nations and indigenous populations across the globe, human–nature relationships imply a more balanced relationship between human beings and ‘mother nature’ (Kimmerer 2013; Melo 2013), based on principles of complementarity, reciprocity, kinship and communal labour (Mayer 2005). These include respect for nature’s cycles and engaging with nature in a reciprocal relationship, which implies at the same time ‘raising’ and ‘being raised’ by nature (Van den Berg 1990). These guiding principles are reflected through everyday practices and governance structures, which deeply link nature and community life, and are also reproduced in the urban scenario (Hernández- García and Caquimbo-Salazar 2018).

There seems to be broad agreement on the need for locally adaptive NBS, tailored to specific contexts and challenges, including the wide range of governance arrangements, political regimes, socio-economic factors, as well as cultural and historical considerations prevalent across both the GN and the GS (Davies and Lafortezza 2017; Kauark-Fontes et al. 2023). However, it is notable that countries in the GN often possess more substantial financial resources for NBS due to their higher income levels and well-established environmental policies (Kauark-Fontes et al. 2023). Conversely, this financial advantage is not typically enjoyed by countries in the GS, leading them to encounter challenges in securing funding for large-scale NBS projects (Castelo et al. 2023; Kauark-Fontes et al. 2023). Furthermore, policy support for NBS in the GS tends to exhibit greater variability, or being completely absent, resulting in difficulties when trying to coordinate efforts across diverse government agencies (Breen et al. 2020). Moreover, there are major challenges that may ultimately impact the successful implementation of NBS in Latin America, such as weak local government structures, informal settlements, significant socio-economic inequalities and conflicts with indigenous community practices (Breen et al. 2020; Portugal Del Pino et al. 2020; Kauark-Fontes et al. 2023).

Taken together, the core critiques and practical problems faced in developing urban NBS suggest a need for continued debate and development of both the concept and practice of NBS, exploring how they could provide more effective responses to contemporary socio-ecological challenges in the cities. The introduction of NBT is part of the rich, contemporary debate around NBS, in line with the socio-ecological, systemic and pluralistic approaches discussed above. We present its main premises in the next section.

### Nature-Based Thinking (NBT)

**Fig. 1:**
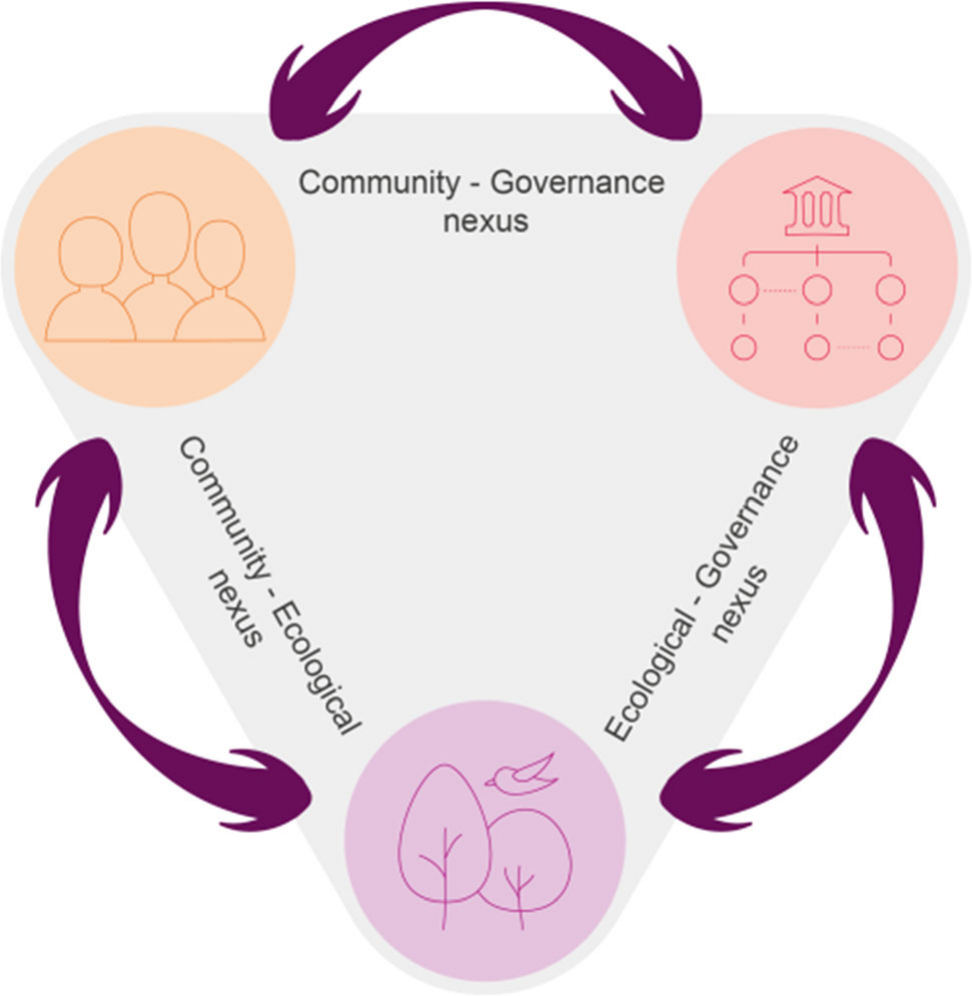
Nature-Based Thinking incorporates three dimensions and their relations, as an approach or a mindset to examine the human–nature interaction in NBS development. These relations are reciprocal and interdependent, being all of the same weight (and importance) (Adapted from: Randrup et al. 2020)

NBT is proposed as a mindset that sees nature and humanity as indissolubly connected, working across sectors, disciplines and levels of governance to implement NBS over conventional infrastructure, whilst advocating and educating for change that supports this transformation (Wild 2020). NBT attempts to incorporate a relational and reciprocal perspective on human–nature relations (see Fig. 1) proposing three inter-related dimensions:

(i) *nature itself and its ecological processes,* including securing room for nature beyond services and solutions, and especially in urban areas, to build in more space for natural processes, ecosystem functioning, and long-term unpredictability, whilst recognising nature’s intrinsic values; e.g.,: the inherent worth and right to exist that is attributed to ecosystems and species, regardless of their usefulness to humans. (ii) *The institutions formally, or informally owning, governing and/or managing a natural space*, recognising the need to break siloes and build opportunities for linking formal government with local communities through cyclical process to plan, design, construct, and manage different types of urban green spaces; and, (iii) *the communities living in, for and with nature*, to reconnect urban populations with nature directly, physically as well as spiritually and emotionally, by expanding the opportunities for urban populations to experience the unpredictability and heterogeneity of nature.

The relations between the dimensions are just as important as the dimensions themselves, and are represented by three nexus: (i) The nature-governance nexus, which relates to how nature is being developed, governed, managed and maintained; (ii) the community-nature nexus, which describes how nature relates to communities living in, for and with nature; and (iii) the community-governance nexus, which describes how institutional governance structures engage and involve citizens in NBS processes but also, how citizens, often independently (or even in conflict with) governments interact with nature.

In essence, NBT poses a shift from a solutionist to a systemic approach, by suggesting that the direction and magnitude of the changes that cities make could ultimately be defined by how the links or interactions among the three dimensions generate or mitigate social-ecological problems. This would again depend on the extent to which cities can maintain and deal with all three connections simultaneously. The NBT framework triggers questions about how to reach a balance between the three nexus. For example, in terms of the *nature-governance nexus*, one emerging question is how the value of NBS and their services can be formally acknowledged within urban governance, whilst recognising nature’s intrinsic value. In terms of the *community-nature* nexus, it forces us to question how culturally diverse and community-centred ways of relating to nature which have been crucial for survival in economically disadvantaged regions can be preserved and integrated into urban development. Finally, the *community-governance* nexus sparks questions about which new practices, technologies and governance approaches are needed to co-create long-sighted social-ecological transformations.

Next, we describe the process by which we have collaboratively explored how a NBT perspective can broaden and expand NBS frameworks.

## Materials and methods

This paper is based on a reflective and iterative research process within the EU funded Horizon 2020 CONEXUS project (Research & Innovation Action). CONEXUS is a cooperation between European and Latin American cities, starting in 2020, and implementing NBS pilots within ‘Life- Labs’ (urban living labs) in seven cities: four in Latin America (Bogota, Colombia; Santiago, Chile; Sao Paulo, Brazil and Buenos Aires, Argentina), and three in Europe (Lisbon, Portugal; Barcelona, Spain and Turin, Italy). In Table 1, the key NBS for each city are listed as a context for the NBT discussions.

**Table 1:** Key NBS implemented by each of Conexus cities in the project ‘Life-Labs’

City	NBS purpose
Bogota	Structural and functional restoration of local streams, upstream riparian forest restoration for peri- urban sustainable urbanisation
Barcelona	Improving biodiversity and environmental performance in urban allotment gardens
Buenos Aires	Restoring wetlands for storm water phytoremediation, establishing Sustainable Urban Drainage Systems (SUDS), daylighting of culverted rivers, and green fences to ameliorate urban air pollution
Lisbon	Enhancing ecological connectivity and demonstrating place-keeping principles
Santiago	NBS for air quality and flooding as well as addressing environmental justice issues
Sao Paulo	Habitat effects on human wellbeing and climate change mitigation (thermal comfort, CO2 reduction and pollution reduction)
Turin	Repurposing public areas, establishing sustainable urban drainage systems (SUDS), increasing urban biodiversity

We compile viewpoints from both the Global North (GN) and the Global South (GS), a crucial dialogue that has long been demanded and is essential for overcoming the global environmental crisis. Different voices and discourses from academia, practitioners and citizens in European and Latin American cities implementing NBS are thus represented in this paper.

**Fig. 2:**
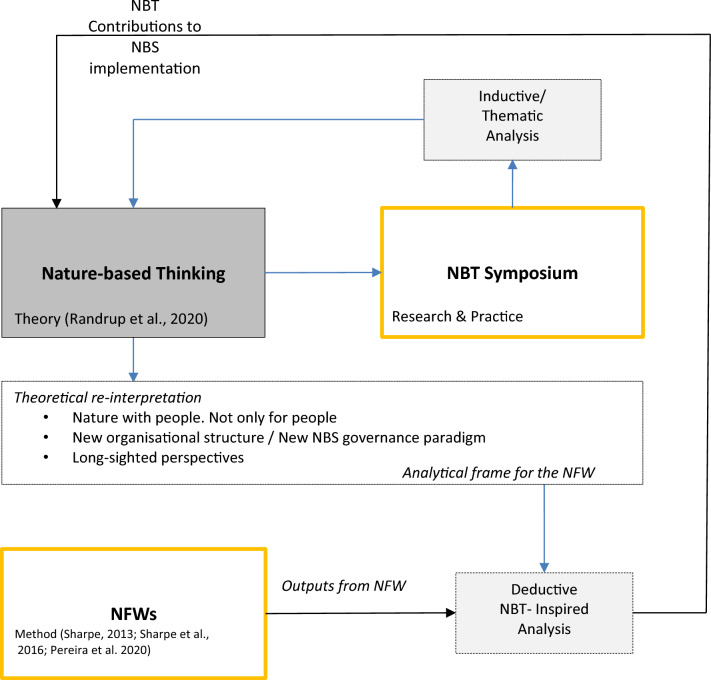
Iterative process for the analysis of the Nature-Based Thinking (NBT) Symposium and Nature Future Workshops (NFW) results, the blue lines signal the NBT symposium feedback into NBT and then into the NFWs. The dotted boxes signal the different types of analysis performed on both Symposium and NFW results

The reflective, iterative research process is in line with qualitative research principles, where the role of iteration contributes to build on a deeply reflective process, developing meaning through a series of iterations between data generation, analysis and reflection processes (Srivastava and Hopwood 2009). As part of the reflective process, we organised a transdisciplinary symposium within CONEXUS, to discuss the concept of NBT, followed by a series of Nature Future Workshops (NFWs), which were held in six of the seven cities. The results from the NBT Symposium were used as an analytical lens to analyse and reflect on the NFW results. This process is described in more detail in Fig. 2.

A description of each of the reflective events is presented next.

### Nature-Based Thinking symposium

A symposium was organised to deepen understandings of NBT and to identify possible synergies, overlaps or challenges for the cities. A call for abstracts emphasised five major themes about NBT: (1) New practices, technologies and governance for long-sighted social- ecological transformations; (2) Concepts and related value frames to strengthen human–nature connectedness; (3) Changes to ensure that nature-based approaches become mainstream; (4) Value of nature beyond solutions and services and (5) Transdisciplinarity.

The symposium was held online in December 2021, presenting eight papers which covered the above-mentioned themes. Ninety-four delegates participated, distributed as follows: science/research—36; city/regional governments—16; SME/business—14; NGO/civil society—10; policy/government—17; other—1. Presenters’ profiles were transdisciplinary with both academic and practitioner representation. To motivate reflection, the symposium was organized into two forty-minute sessions, each showcasing the presentation of four papers, followed by a dynamic panel discussion involving two pre-assigned paper reviewers, the authors, and the audience.

Based on the outcomes of the symposium, we developed a deeper understanding of Nature-Based Thinking, and thoughtfully integrated these insights back into the foundational principles of NBT, set out by Randrup et al. (2020). This process involved transcribing the symposium talks and panel discussions, followed by a rigorous thematic analysis. The initial analysis was conducted by four co-authors, and the outcomes were subsequently reviewed and summarized by two additional co-authors, culminating in the identification of three major overarching themes:

- Nature *with* people instead of nature *for* people
- New organisational structures/NBS governance paradigms
- Long-term perspectives

These themes are further described in "Nature with people instead of nature for people" section.

### Nature Futures Workshops

The purpose of the Nature Future Workshops (NFWs) was to invite leaders, experts and agents of change, working with or around NBS to (i) jointly explore nature-based futures that support the wellbeing of all life; (ii) engage a range of perspectives and plurality of voices in exploring desired futures for nature (and life) and (iii) promote mutual transformative learning and seeds of transformative change through a richer and more connected understanding of nature and life’s potential. The NFWs methodology was based on the three horizons framework (3-H) (Sharpe 2013; Sharpe et al. 2016; Pereira and Bina 2020). Futures methods like 3-H are not concerned with forecasting specific futures but with opportunities for reflection on the present and the futures we are currently creating, alongside creative exploration of preferred futures. The process reveals gaps between current actions and what is needed to realise preferred futures, generating critical reflection on the changes we need to make. By considering concrete actions required to realise aspirations, the method focuses on taking action to change current trajectories.

NFWs were developed collaboratively with Life-Lab coordinators in six of the seven CONEXUS cities. One of the cities had carried out similar and extensive workshops very recently, and therefore decided not to carry on the NFW. A guidance document was produced to support each city. Due to varying COVID-related restrictions, both online and in-person versions were held between January 2022 and June 2022. Life-lab coordinators identified and invited a diverse range of participants aiming to get a ‘mixed view’ on the future of cities. In total, eighty-eight delegates participated in the workshops: science/research—42; city/regional governments—32; SME/business—5; NGO/civil society/activists – 6; other—3.

To foster reflection, participants answered a preparatory survey before the NFW. The first part presented open questions where participants described three current trends affecting nature in their city, three key drivers of change and the three promising seeds of change. They were also invited to write a postcard from their future selves, describing (and imagining) their cities in 2050. Results from the preparatory survey were presented at beginning of workshops to start the reflections and discussions. In the workshop, the first step *“The futures we’re making now—Horizon 1*” aimed to build a narrative about the futures participants are making now (including NBS interventions). Participants projected current trends, surveyed the horizon and contemplated the futures that would unfold in their cities if 'business as usual' approaches persisted. The second step “*The futures we want—Horizon 3”* aimed to explore and develop shared visions of desired futures. Finally, a ‘back casting’ third step *“How we can get there – Horizon 2”* sparked reflection on possible pathways from desired futures to the present, considering key actions and interventions.

Following each workshop, a comprehensive report detailing the survey results and workshop discussions was generated. This was succeeded by reflective meetings with Life-Lab coordinators and other local participants, providing an opportunity for in-depth discussions to validate and refine the data. The main question guiding these meetings was whether the NFWs results were aligned to the NBT themes identified during the symposium and the degree to which this alignment was evident.

Core insights that emerged from the NFWs were grouped according to the three central perspectives derived from the NBT Symposium: 1) nature *with* people, instead of nature *for* people, (2) the importance of building new governance structures and (3) the need for long-term perspectives. Data resulting from the NFWs cannot be read as offering objective assessments of possible futures, rather our reading of the three horizons sought to draw common themes related to the three dimensions of NBT.

## Results

In this section, we present the results both from the NBT symposium and the NFWs. As stated in "Nature-Based Thinking symposium" section, the three emerging themes from the symposium guided the analysis of the future workshops results. This section is thus structured around those three themes. Illustrative quotations are taken both from the symposium and from workshop reports and participant statements from pre-workshop surveys. Note that quotes written from a future perspective are marked with an asterisk (*) at the end.

### Nature with people instead of nature for people

#### Symposium results

Participants in the symposium agreed that current challenges and crises cannot be sufficiently addressed within the dominant political-economic systems and concurrent worldviews where humans and nature are detached. In both GN and GS cities, there is a need to build a new relationship between people and nature, less utilitarian and more inclusive.

*“…the challenge is how to change, us individuals but especially as communities, as collectives, our mindsets regarding the relationship with nature…”* (NBT symposium, 12/01/2021).

Non-hegemonic and non-Western forms of knowledge and logics framing urban life and values need to be rescued, protected and re-incorporated in planning, management and governance for the transformation of cities. These include intrinsic and relational values of nature, and action going beyond purely instrumental conceptions of nature’s value. Participants from cities in the GS have emphasized the importance of reevaluating alternative socio-environmental epistemologies. These encompass diverse ways of understanding and engaging with nature, exemplified by indigenous and ancestral knowledge and cultures. This entails embracing indigenous and ancestral knowledge and cultures, as well as learning from the ways in which local and indigenous communities in the GS continue to foster meaningful connections with nature and organize their lives around it.

*“…communities have their own tradition and knowledge, linked with different relationships with nature, and this can be very helpful in thinking about this shift from NBS to NBT […] to see what in the past, in relation to ancestral and indigenous knowledge in Latin America, in Andean countries in particularly, can be put up today”* (NBT symposium, 12/01/2021).

By acknowledging and integrating these traditional practices, it could be possible to enrich our understanding and make our approaches to environmental challenges more pertinent.

#### Nature future workshops results

The visions of the future demonstrated a common desire to reshape societal relations with nature, including the granting of more space to nature across metropolitan regions. This was powerfully evident in images of strategically planned natural corridors, enabling native flora and fauna to flourish in expanded and protected habitats, including ecosystem restoration processes. Connection was a core theme in all workshops, describing both a perceived lack of physical connectivity between green spaces as well as a sense of alienation between society and nature in the present. A desire to overcome it through the creation of new ways of living and relating, better attuned to natural rhythms and cycles was manifest, for example, through local food production:

*“We developed a more local and organic food production chain, with agroforestry spaces spread throughout the city, producing healthy and poison-free food in backyards and public gardens. Much of this work is carried out by cooperatives, using open areas more collectively…”* (Sao Paulo Workshop, 02/06/2022)*.

*“Today we are clearly aware that the best solutions are obtained with nature as an ally and respecting traditional communities and the regional aspects of each culture…”* (Sao Paulo Workshop, 02/06/2022)*.

*“My neighbourhood still has many green spaces, but now people understand the relationship with nature in another way; the species are adapted to the local climate to avoid the waste of water in irrigation, herbicides are no longer applied ”* (Lisbon Workshop, 05/04/2022)*.

At the same time, some participants also pointed to key tensions and trade-offs that might be involved in ‘giving back’ urban areas to nature. Some were keen to ensure that the city would remain recognisably a city and not become rural (Lisbon), or were concerned about potential challenges of living with a wilder urban nature. What if, for example, native wild animals like pumas (Spanish for Cougar, e.g., *Puma concolor*) were reintroduced to the areas surrounding cities like Santiago or Buenos Aires?

Perhaps most profoundly some participants, particularly in Latin America, expressed concerns that interventions prioritising nature’s needs rather than people’s, in regions where basic needs are still not being met, were a privileged agenda:

*“…without covering basic needs, we cannot think in a context based on nature.”* (Buenos Aires Workshop, 11/05/2022).

Reinforcing this point, visions of nature-futures across all cities revealed varying desires for wider transformations in economic and social life to give people the resources, time and space to live differently with nature. Various paradigms and proposals to make this happen were explicitly referenced in the workshops, including ideas like universal basic income, de-growth and circular economies. This reflected a sense that most people do not have the material conditions they need to rework their relations with nature. It also suggests the importance of considering how NBT might work within or alongside other agendas or social movements to generate change, rather than remaining narrowly focussed on NBS:

*“Somewhere along the way, there was a profound rethinking of the ways we use our time. Instead of working around the clock to make ends meet and borrowing to support unsustainable consumption, people now value the many little things that give them satisfaction. […] The time for all of this was made possible by the introduction of a universal basic income (UBI) freeing people from wage oppression to make more positive choices about how to live…”* (Lisbon Workshop, 05/04/2022)*.

*"...it is necessary to go through processes of awareness-raising and education… where the slowing down of growth is no longer seen as negative."* (Turin Workshop, 15/06/2022).

### New organisational structure/NBS governance paradigm

#### Symposium results

Another emerging theme on the symposium was the focus on organisations as the main driver for NBS, and ultimately the identification and involvement of people in NBS planning, management and evaluation as crucial for the long-term success of NBS and changing mindsets towards nature.

*“We feel that governance is a very important aspect of understanding pathways to mainstreaming NBS […] these days is not the government deciding how cities are going to look like, there is a range of actors influencing this and governments are more and more working in partnership and are also lobbied a lot by other actors to change what they are doing. So they are not the only driving force of societal change.”* (NBT symposium, 12/01/2021).

Multiple new and alternative governance approaches are needed to collectively produce and share knowledge and experiment with NBS. These approaches need to recognise transdisciplinarity and plurality, and to integrate different perspectives, values, expectations and knowledge cultures from local communities.

*“…the agenda of plurality and diversity, taking from biodiversity but linking to ideas of knowledge systems and diversity, and the cultural diversity behind different knowledge systems and the richness that they can bring in […] respect of different cultural perspectives and epistemologies is certainly central”* (NBT symposium, 12/01/2021).

#### Nature future workshops results

If reconnecting to nature was a commonly articulated desire across the NFWs, there was also a consistent emphasis on the need to reshape relations between citizens and governing institutions. Reflecting long-standing debates within governance and planning, there was a widely shared sense of disconnect between people and decision-makers and a marked problem of mistrust between people and government:

*"... Local Governments learned to share their difficulties and challenges with citizens, and in this mutual learning much was revealed, leading to greater mutual understanding, and the willingness to help and collaborate towards the Shared Vision."* (Lisbon Workshop, 05/04/2022)*.

A persistent critique of ‘business as usual’ approaches to NBS was that they operated at a technical level that failed to engage a diverse enough range of voices, perspectives and ways of knowing, especially those of socially marginalised groups and, perhaps more radically, of other-than-human actors:

*"The future, even if developed around a technological framework, must therefore take into account the needs of nature and the relationship of nature with the human being. In 2050, this was recognised, and with the awareness of our past mistakes, it was possible to stop wanting to overcome natural limits. Now science supports and helps us: “Nature always wins. We are good at discovering and inventing things, but we still don't know how to live without nature, since we cannot imitate it."* (Turin Workshop, 15/06/2022)*.

Given the participants involved in the NFWs were largely actors working in, or closely related to governing institutions, it is perhaps unsurprising that this was sometimes seen as a problem of a lack of public awareness and education, requiring a focus on wider processes of social learning about nature and its governance. However, it was also widely acknowledged that governance processes remained remote from citizens’ lives, too often doing things to people not with them, and failing to integrate knowledge of local needs and conditions. It was widely acknowledged that this particularly affected the least well-off, intensifying existing inequalities. Across several NFWs, it was also felt that the technocratic frame of NBS did not typically do enough to emphasise the educational potential of nature-based interventions.

Participants’ images of a desired future persistently emphasised how this disconnection and mistrust would be overcome, often in a more ‘localist’ frame in futures where people had time to actively participate in both inclusive local governance processes and the management of their own environment:

*“The result of this change in trajectory was, in my opinion, the new social and environmental collectives that grew throughout the city, the change on public policies and planning, and the spaces for co-creation and construction that were conceived to support citizen participation in decision making. I hope we continue having these spaces and building the city from local territories.”* (Bogota Workshop, 08/03/2022)*.

For some, these new relations would be infused with a new ethos of care, both for nature and for the people, places and lives affected:

*“...then, Nature-Based Solutions, promoted as solutions for the future, focused on developing local knowledge and practices to care for nature … involving communities - from the youngest to the oldest.”* (Lisbon Workshop, 05/04/2022)*.

Some other participants saw signs of hope for the future in the insurgent practices of citizens appropriating and managing local natural spaces for themselves as a means of pressing for institutional change and demanding more collaborative and responsive modes of governance:

*"With more support for communities to take control of their own green space’s communal gardens, parks, forests and farms began to appear everywhere. When they saw that they could really influence things people began to trust the process and others saw what was happening and wanted to get involved too."* (Santiago Workshop, 11/01/2022)*.

### Long-term perspectives

#### Symposium results

Long-term planning and contextually appropriate designs are basic needs for the development of nature. Apart from this being an ecological aspect, there is a need to identify all stakeholders involved in any NBS process, and seek to secure their long-term engagement, aiming for a cultural change towards more sustainable socio-natural relations:

*“We can see great potential for local people to apply deep ecological knowledge not only to design, but through long-term stewardship, maintenance and management of green space and networks.”* (NBT symposium, 12/01/2021).

The need to work particularly with children and the young was stressed, aiming for long-term results, but also because young people can act as innovative ambassadors of transformative thinking, inspiring adults.

#### Nature future workshops results

Related to insights about participatory governance, another core desire articulated in all NFWs was to see people more actively involved in the long-term ownership, management and maintenance of their natural spaces (see also *place-keeping*, Wild et al 2008; Dempsey and Burton 2012). This was seen as key to generating stronger communal bonds, connecting people to one another and to nature in new ways and building cultures within which people learn to care for one another and the natural world in new ways:

*“In 2050, we are experiencing the inverse of globalisation, as we now give more importance to the local scale (…). This created a greater local identity, and the potential for cooperation and collaboration between people. With the appreciation of public space on a local scale, we have created more socially integrated open areas, and started to incorporate these spaces into our daily lives.”* (Sao Paulo Workshop, 02/06/2022)*.

However, a further core contribution from the NFWs was to reflect on the role and value of such utopian-sounding aspirations in thinking about nature-based futures. In this regard, it is worth noting that speculative and disruptive thinking about the futures we want was met with skepticism by some participants who questioned taking time for speculative workshops. Many others found it hard to escape from dominant and largely pessimistic images of the future that framed their thinking:

*“At the beginning of the discussion about the H3, some participants asked themselves: Do we really have to be optimistic? It was difficult to do so, given the socio- environmental and political scenario in which we operate.”* (Sao Paulo Workshop, 02/06/2022).

Some participants, however, pointed to the possibility of seeing NBS as a form of tactical urbanist intervention, pointing the way towards the wider structural changes they sought to make. For example, in Lisbon, there was a discussion of the role of “guerrilla plantations” (i.e., illegal/ insurgent planting) in shaping a city “inhospitable to the circulation of private cars”:

*“When the roads above the green [metro] line are full of trees instead of cars, we'll know things are going in the right direction."* (Lisbon Workshop, 05/04/2022).

Such radical or evenly openly political ambitions point back to the possibility of considering NBS within a wider frame of the transformations required to create sustainable futures in response to urgent societal challenges. In this way, futures thinking may be considered a valuable part of a broader-based NBT, focussed on pushing beyond the technical implementation of NBS, to consider a longer-term perspective and the broader political-economic and societal transformations it requires.

## Discussion

Through this paper, we contribute to current debate on NBS and the need for more inclusive and pluralistic approaches. Critiques of NBS often suggest a need for a deeper debate on its scope and efficacy; challenging prevailing approaches that focus on immediate, solution-oriented and instrumentally defined issues (Schröter et al. 2014; Eggermont et al. 2015; Bina et al. 2020; Pereira and Bina 2020; Maller 2021), in order to consider more holistic, inclusive and long-sighted alternatives. We consider this paramount in the face of the climate emergency, biodiversity loss and the persistence of social and environmental injustices.

Based on extensive reflection across European and Latin American cities, we have critically interrogated current practices in terms of their underlying assumptions about nature–human relations. In doing so, we have sought to explore how NBT might be developed as an approach to address future city transformations via nature-based interventions whilst cultivating a wider, more long-sighted, locally embedded and inclusive approach in response to the conjunction of crises we face. By making implicit assumptions about nature explicit, and by considering the conceptions underpinning interventions, their effects and how they could be re-thought, NBT could represent a point of departure where different ideas about the role(s) of nature are opened up to debate. We therefore suggest core areas for developing NBT further as a principle for pushing at the limitations of NBS, in particular, three key themes emerged across the discussions we are reporting on here, i.e. new ways of relating to nature; new modes of governing; and long-term perspectives.

### New ways of relating to nature

A relational and reciprocal conception of human–nature relations is needed to ensure NBS promote long-term sustainability and to move away from anthropocentric, functionalist and solutionist perceptions. To limit the persistent impacts of human activities on natural systems, as well as to contain the risk from multiple crises (UNEP 2021), far-reaching transformations of human–nature relations are required. To transform dominant ways of thinking about nature, transformative thinking is needed (Frantzeskaki et al. 2017), including the ability to imagine a transformed world and to anticipate how those transformations can be brought about. NBT as a process (and mindset) orientated towards transformative thinking cannot be defined a priori in static terms, on the contrary, NBT argues for the embedding of NBS in the local contexts where these are being implemented. As the long history of sustainability has shown, it is at the local level that several underlying tensions and trade-offs are revealed. It is also at the local level, as some of our NFWs have shown, that the pursuit of new ways of thinking about human–nature relations can raise questions of privilege and the all-too-often cast aside issues of unjust and unequal relations. This is especially evident when comparing the GN and GS challenges in NBS implementation: during the NFWs, one of the recurring concerns from Latin American participants seemed to be the need for prioritizing survival before caring for nature (i.e. see quote on “Nature with people instead of nature for people" section) which points to the need of addressing latent issues of inequality and poverty in the region, and how these might be affected through NBS implementation (Anguelovski et al. 2019; Breen et al 2020). Given well-evidenced demonstrations of, for example, green gentrification in cities (Anguelovski & Corbera 2023), it is crucial that any ‘fuzzy’ (vague) promises of social, cultural, economic and environmental benefits are carefully interrogated when determining local priorities for urban NBS interventions.

More broadly, new human–nature relations call for new ways of organising daily life, which in turn require new possibilities predicated on wider socio-economic transformations. The appeal for more ‘time’ in our lives (see i.e. quote on "Nature future workshops results" section) to enable us to engage differently with (as part of) nature is an emblematic illustration of how deeply dominant systems would need to change such as, through the introduction of a universal basic income (UBI) and ideas of de-growth. This highlights the need to shape locally sensitive, plural and dynamic understandings of nature-based interventions, where the values of nature respond to key societal priorities but always with a core emphasis on equity. NBT seeks to add to current ways of governing and of relating to nature, by transcending a narrow focus on nature as separate from socio-economic systems and avoiding 'magic-wand like’ solutionist agenda. If we refocus our attention on the need for value-based interventions in the wicked problems found in complex socio-ecological systems, it may help to emphasise the importance of moving beyond a technocratic frame towards a more locally situated and deliberative mode of policy making. Hence NBT’s underlying principles need to be established through value-driven debate about the changes required in different places at different times, supporting wider processes of social learning and communication across actors and sectors. This requires understanding of the power relations, governance processes and networks of actors through which transformations can be developed and realised. It also requires a greater sensitivity to diverse forms of local knowledge and ways of relating to nature, including more explorative approaches that seek to challenge ‘business as usual’ practices and give voice to the voiceless, including more-than-human-perspectives.

### New modes of governance

Formal governance tends to operate through top-down institutional initiatives that struggle to connect with diverse perspectives or people’s everyday lives and spatial practices. Experiences and perspectives from the Symposium and NFWs echoed longstanding calls for more co-designed and co-produced knowledge about our cities, adding voice to growing calls to find ways of including a dramatically wider range of ways of knowing within urban governance, encompassing a plurality of epistemic and ontological premises. Reshaping relations with nature therefore requires the development of new governance structures that can work towards long-standing visions of horizontality, transdisciplinarity and inclusion in the planning, development and management of urban nature. As described above, novel governance structures will be needed to encompass new nature–human relations, but these could also be designed to foster and facilitate changes in these relations. In this respect, NBT has the potential to spark discussion not only about the ideal but also about how to craft adequate institutional structures that can provide support and sustain desired changes in the long term. Approaches to city planning, management and governance as integrated systems with a clear socio-ecological perspective are needed, placing interactions with nature at their centre in ways that look beyond solutionism (Cockerill et al. 2017), resist narrow technological fixes (Bina et al. 2020), and the monetisation and financialisation of nature (Ouma et al. 2018). This points to the challenges of integrating local knowledge into wider social as well as governance systems. This implies creating governance spaces within which alternative but often marginalised worldviews can influence decision-making, building a more flexible and holistic mindset that allows us to depart from anthropocentric and simplistic/reductionist views of nature–human relations. Thus, although an NBT perspective could be applied in any context, it should be measured by its diversity as it argues for the inclusion of local, and diverse ways of knowing that push beyond technocratic solutionism in various ways: connecting nature with people, promoting broader social learning, and engaging more creatively in participatory thinking. Hence, NBS interventions require the prioritisation of social learning and evidence about impacts, seeing those also as part of a much broader systemic transformation.

### Long-term perspectives

Thinking about the long term is crucial for the sustainable management of NBS and challenges standard investment practices that too often neglect the importance of place-keeping and, as a result, the scope for learning and reworking of social relations that can emerge through more collaborative and collective governance of urban space (Kabisch et al. 2016; Wickenberg et al. 2021). At the same time, the scale of the transformations now required to meet contemporary societal challenges means that nature-based interventions need to be seen as part of wider agendas and political struggles over the scope and scale of change, particularly to work for people facing deprivation. This requires reimagining NBS, not as stand-alone projects or technical elements of green growth-based action plans but as tactical interventions in wider processes of socio-ecological transformation. By fostering this wider perspective, NBT might open up wider questions about the criteria by which interventions are assessed as part of strategies for just transitions.

The aim of collectively reflecting on normative futures through the NFWs was to generate ideas for bridging the gap between desired futures (the utopian impulse) and strategic planning and management. Our inability or sometimes unwillingness to explore this crucial space may point to a range of obstacles that are also relevant to the pursuit of long-term NBT: lack of time; a culture of efficiency and effectiveness that easily denies the space for imagining a world with different premises; and fear of exploring desires that might reveal the undesirability of our past and present. For example, if we take the issue of ‘time’, we can ask how much scope is provided to actors involved in planning NBS strategies and implementation for mutual learning, exploring and connecting across sectors and policy areas. We would likely find that far more effort is focussed on developing interventions, with far less available time dedicated to long-term management, learning and discussing across boundaries (Dempsey and Burton 2012; Randrup et al. 2021).

If transformative change on the scale required to respond to societal challenges involves the means to collectively reflect on the changes needed to shape livable futures, then the NFWs suggest forms of futures literacy that are as yet under-developed in debates around NBS and which dominant modes of scientific discourses continue to treat with suspicion. However, in the absence of wider reflection and debate on the horizons towards which societies are working, there is a danger that the ultimate ends of interventions like NBS will remain under-examined, leaving them open to the kinds of criticism outlined at the start of this paper.

## Conclusions

This study used NBT (Randrup et al. 2020; Wild 2020) as a starting point for a theoretical exploration of how NBT may contribute to rethinking and reworking NBS implementation, based on a series of reflective events held within the EU funded CONEXUS project.

The NBT symposium and the series of NFWs together explored the potential of NBT to develop future cities in Europe and Latin America that place nature at their core. The symposium results guided the analysis of the NFWs and their overall analysis identified three key themes which need to be addressed when further developing NBS inspired by NBT: (i) New ways of relating to nature; (ii) New modes of governance (iii) and Long-term perspectives. These three key themes call our attention to the need for (i) relational-reciprocal conceptions of human–nature relations and moving away from anthropocentric, functionalist and solutionist perceptions; (ii) the importance of developing novel governance structures that can foster and facilitate locally embedded processes, which include a diversity of voices and perspectives in NBS development; and (iii) the need for long-term perspectives that allow both for nature regeneration cycles as well as social reflection cycles dedicated to learning and discussing across administrative and socio-ecological boundaries.

Our analytical reflection strived to collect different voices and discourses from academia, practitioners and citizens representing European and Latin American cities. By conducting both a transdisciplinary symposium and locally based NFWs we have strived to include those voices in our reflection process; however, these should be contrasted with, for example, other studies in similar scenarios that could validate our findings and conclusions. Further studies are required to concentrate on the disparities in NBS implementation between cities in the GN and GS. Additionally, it is essential to examine the intricate human–nature relationships and local knowledge that have so far contributed to the resilience of marginalized communities. This understanding can guide us in co-creating a more sustainable urban future in harmony with nature. We propose additional case studies that delve into the three nexus of Nature-Based Thinking (NBT) in NBS implementation. Such studies should encompass small and medium-sized cities, which are anticipated to be the metropolises of the future, as well as cities located in the Global South.
